# Randomized, placebo-controlled, double-blind trial of Swedish snus for smoking reduction and cessation

**DOI:** 10.1186/1477-7517-8-25

**Published:** 2011-09-13

**Authors:** Gordana Joksić, Vera Spasojević-Tišma, Ruza Antić, Robert Nilsson, Lars E Rutqvist

**Affiliations:** 1 Vinča Institue of Nuclear Sciences, University of Belgrade, Belgrade, Serbia; 2 Academic Association for Research on Occupational and Public Health (AROPH), Zemun-Belgrade, Serbia; 3 Swedish Match AB, Maria Skolgata 83, 118 85 Stockholm, Sweden

**Keywords:** Randomized trial, double-blind, placebo-controlled, Swedish snus, smoking reduction, smoking cessation

## Abstract

**Background:**

Epidemiological studies suggest that smokeless tobacco in the form of Swedish snus has been used by many smokers in Scandinavia to quit smoking, but the efficacy of snus has so far not been evaluated in controlled clinical trials.

**Methods:**

We conducted a randomized, double-blind, placebo-controlled, clinical trial aimed at assessing the efficacy of snus to help adult cigarette smokers in Serbia to substantially reduce, and, eventually, completely stop smoking. The study enrolled 319 healthy smokers aged 20-65 years at two occupational health centers in Belgrade, Serbia. Most of them (81%) expressed an interest to quit rather than just reduce their smoking. Study products were used *ad libitum *throughout the 48-week study period. The main study objective during the first 24 weeks was smoking reduction. The primary end-point was defined as a biologically verified reduction of ≥ 50% in the average number of smoked cigarettes per day during week 21-24 compared to baseline. During week 25-48 participants were actively instructed to stop smoking completely. Outcome measures of biologically verified, complete smoking cessation included 1-week point prevalence rates at clinical visits after 12, 24, 36, and 48 weeks, as well as 4-, 12- and 24-week continued cessation rates at the week 36 and 48 visits.

**Results:**

At the week 24 visit, the proportion of participants who achieved the protocol definition of a ≥ 50% smoking reduction was similar in the two treatment groups. However, the proportion that reported more extreme reductions (≥ 75%) was statistically significantly higher in the snus group than in the placebo group (p < 0.01). The results for biologically verified complete cessation suggested that participants in the snus group were more likely to quit smoking completely than the controls; the odds ratio (snus versus placebo) for the protocol estimates of cessation varied between 1.9 to 3.4, but these ratios were of borderline significance with p-values ranging from 0.04-0.10. Snus was well tolerated and only 2/158 (1.3%) participants in the snus group discontinued treatment due to an adverse event (in both cases unrelated to snus).

**Conclusions:**

Swedish snus could promote smoking cessation among smokers in Serbia, that is, in a cultural setting without traditional use of oral, smokeless tobacco.

**Trial registration:**

www.clinicaltrials.gov, identifier: NCT00601042

## Introduction

The smoking prevalence is substantially higher in Central and East European countries than in West Europe [1]. In Serbia, for instance, smoking prevalence among both males and females is reported at 30-40% [2]. During recent years, Serbian public health authorities have initiated antismoking campaigns but the funding for such activities is limited, as well as for modern, pharmaceutical smoking cessation products. Progress is also hampered by the relatively low public awareness of the health hazards associated with smoking.

Sweden demonstrates a unique pattern in terms of smoking-related disease; male smoking-related deaths are radically fewer than in other European countries, and Sweden is also the only EU country where male smoking prevalence is lower than among females [3]. During recent decades smoking among Swedish males has decreased to a larger extent than among women, probably related to the prevalent use in men of snus, a traditional Swedish oral tobacco product, as a smoking cessation aid and replacement for cigarettes [4]. The use of low-nitrosamine smokeless tobacco of the Swedish type is associated with health risks that are only a fraction of those caused by smoking [5-7].

Use of nicotine replacement may promote smoking cessation as evidenced by numerous controlled clinical trials on the role of pharmaceutical nicotine products [8]. However, the cost of such products is prohibitively high for most Serbian smokers. Swedish snus offers another possibility for nicotine replacement. The Swedish experience provides strong indirect support to the notion that snus can promote smoking cessation and help to reduce tobacco related disease [9-12]. Snus is also generally regarded as less harmful than other smokeless tobacco products [13].

In contrast to Scandinavia, Serbia has no tradition of oral, smokeless tobacco. Therefore, a pilot study was conducted in Belgrade during 2004-2005 where 21 smokers tested different Swedish snus products. The main objective was to assess the acceptability of snus in a Serbian setting. A marked reduction of average carbon monoxide levels in exhaled air at the end of the one month test period indicated a substantial reduction of the number of cigarettes smoked. The study also demonstrated that, if properly flavored, an oral moist tobacco product like Swedish snus may be acceptable to both male and female Serbian smokers.

Recruitment to a smoking cessation program may be more successful if the proposed goal is to reduce smoking rather than total cessation. Smokers who have made previous unsuccessful quit attempts might abstain from participating in a program if the requirement is immediate, total abstention. Initial smoking reduction may facilitate complete cessation later on [14].

These circumstances constituted the rationale for the randomized trial presented here. The study aimed at assessing the efficacy of a traditional Swedish low nitrosamine smokeless tobacco product (snus) to help adult cigarette smokers in Serbia to substantially reduce their smoking and, eventually, completely stop smoking.

## Methods

### Study design

This study was an investigator-initiated, randomized, multi-center, double-blind, parallel-group, placebo-controlled, phase 4 clinical trial. It focused on the potential of Swedish snus to reduce smoking and increase quit rates among cigarette smokers motivated to reduce their smoking or quit completely. The study was conducted in compliance with the ethical principles of the Declaration of Helsinki and the International Conference on Harmonization Good Clinical Practice Guidelines (ICH-GCP) at two occupational health care centers in Belgrade, Serbia during January 2008 through March 2010 [15]. The study was approved by the centers' institutional review board, and all participants provided written informed consent prior to entering the study. Before initiation, the study was registered on http://www.clinicaltrials.gov (identifier: NCT00601042).

### Study population

Participants were recruited through posters and other printed material distributed at or in the vicinity of the study sites, and by word-of-mouth. The two sites were occupational health centers located at the head office of a large Serbian corporation (NIS-Jugopetrol) and at a major research institution in Belgrade (Vinča Institute of Nuclear Sciences). The inclusion criteria were: age between 20 through 65 years, history of daily smoking for more than one year, an average daily consumption of more than 10 cigarettes during the past month, motivation to substantially reduce or quit smoking, good general health, and acceptance not to take pharmaceutical nicotine products or any other non-protocol treatment to facilitate smoking cessation during the study period. Exclusion criteria were: uncontrolled hypertension (systolic > 140 mg Hg, diastolic > 90 mg Hg), history of coronary heart disease, other significant heart condition, or any medical condition that may interfere with study procedures, pregnancy or nursing, current abuse of alcohol or illicit drugs, current active oral disease that may interfere with use of study product, significant current psychiatric disease or psychosocial problems that may interfere with study procedures, and use of pharmaceutical or other products for smoking reduction or cessation within the past 3 months.

### Study products

The products were manufactured by Swedish Match AB according to the GothiaTek^® ^standard [16] and were supplied in identical, food-grade, plastic containers. The products came in sachets (pouches) that were placed in the anterior part of mouth between the upper gingiva and cheek for 30-60 minutes. The participants could choose from two different sachet sizes (0.5 g and 1 g) and two different flavors (liquorice and eucalyptus). Swedish snus according to the GothiaTek^® ^standard is a low nitrosamine, moist, oral tobacco product with a water content of c. 45-55%, a nicotine content of c. 1%, and a pH of c. 8.5. The nicotine uptake from snus sachets in comparison with a 2 mg nicotine polacrilex gum was described previously [17]. Snus was found to provide a more rapid uptake than the gum. However, the nicotine uptake from smoked products such as cigarettes is much more rapid than with oral tobacco due to the pulmonary mode of delivery [18].

The placebo snus products were almost identical to the snus products in physical appearance, mouth feel, pH, flavoring, and other sensory characteristics but they did not contain tobacco or nicotine.

### Interventions

With stratification by center, and using a block size of six, a predefined, central, computer-generated randomization sequence assigned participants in a 1:1 ratio to receive snus or matching placebo. Randomization was done by consecutively associating each included participant's identifiers with a unique, computer-generated sequential number. Lists at the study sites linked these numbers to specific study products, that is, snus or placebo. At the sites all study products were identified solely by identification numbers which ensured that both participants and investigators were blinded to treatment assignments. The protocol did not include procedures to assess the success of the blinding.

Each participant was scheduled to be followed for a total of 48 weeks. Study products were distributed to the participants during the entire study period. Participants were instructed to cut down on smoking as much as possible or quit smoking completely. Whenever they felt an urge to smoke, they were instructed to take a sachet of their allocated product. The number of sachets consumed per day was determined by the participants themselves. There was no prescribed tapering of product usage. Unblinding of treatment assignments was not done until after a participant had concluded the entire 48 week study period. Those who wanted to continue with snus after 48 weeks were obliged to buy commercially available products.

None of the study centers had previous experience with smoking cessation interventions (as quit smoking programs were non-existent in Serbia at the time of the trial) or smokeless tobacco (as there is no traditional use of such products in Serbia). However, the trialists attended training sessions prior to the initiation of the study which covered alternative approaches to smoking cessation, the chemical composition of snus, the epidemiology of snus use in Sweden, health effects of nicotine and snus versus cigarette smoking, how to use the study products, the need for adequate nicotine dosing to suppress cravings, methods for counseling of smokers who want to quit, and proper use of study equipment.

Potential participants were invited to seminars during which information was provided about health risks associated with smoking and available smoking cessation strategies. The physiological effects of nicotine were outlined, and an account given of the Swedish experience with snus including potential health risks associated with smokeless tobacco products. A few days-weeks after a seminar, those interested to participate were invited to a baseline visit during which their eligibility was determined and written informed consent to participate was obtained. All included participants were provided with their allocated study product at the baseline visit.

During the first 24 weeks the main study objective was to substantially reduce smoking so the participants were instructed to replace as many cigarettes as possible with their allocated study product or quit completely. They were informed that complete cessation should be the ultimate goal but that smoking reduction could be an important first step toward that aim. Information was given that one 1.0 g sachet used according to the instructions roughly should be able to replace one cigarette. All participants were encouraged to remain in the trial, attend all visits, and complete all assessments irrespective of study product usage or intensity of smoking. The participants were instructed to document on a weekly basis in a diary how many cigarettes they smoked on average per day, and how many study products they had used. Those who managed to achieve the protocol definition of a substantial smoking reduction at the week 24 visit or who had quit completely (see "Study end-points"), continued in the trial up to 48 weeks. During week 25-48 they were actively instructed to quit smoking completely. Participants who did not meet the protocol criteria for smoking reduction at the week 24 visit were counted as treatment failures in all efficacy analyses and were not actively followed after week 24.

### Clinical visits

The baseline visit was followed by 9 clinical visits over a total of 48 weeks. Participants were also contacted by telephone on two occasions (after 1 and 9 weeks). Each follow-up clinical visit comprised protocol assessments, a check of the participant's diary information, assessment of adverse events, and brief counseling (< 5-10 minutes).

### Assessments

The assessment at the baseline visit included medical history, history of smoking including previous quit attempts, measurements of height and weight, blood pressure, CO in exhaled air (Bedfont Micro Smokerlyzer, Sittingbourne, U.K.), assessment of nicotine dependence with the Fagerström Test for Nicotine Dependence (FTND) [19], pulmonary function tests (EasyOne Spirometer, ndd Medical Technologies, Zurich, Switzerland), and blood samples to assess the following biomarkers: total leukocytes (S-WBC), C-reactive protein (S-CRP), total S-cholesterol, high density lipoprotein (S-HDL), low density lipoprotein (S-LDL), S-fibrinogen, and S-cotinine.

Follow-up clinical visits were scheduled after week 2, 6, 12, 18, 24, 30, 36, 42, and 48. They included assessment of CO in exhaled air, self-reported smoking status and study product usage based on the participant's diary information, adverse events, and blood pressure. The pulmonary function tests, blood tests and measurement of weight were repeated at four of these visits (week 12, 24, 36, and 48). The FTND was administered at two follow up visits (week 24 and 48). The results were only considered relevant for those who reported continued smoking.

The telephone contacts scheduled after 1 and 9 weeks included assessment of self-reported smoking status, study product usage, and adverse events.

### Study end-points

The primary end-point was smoking reduction at 24 weeks defined as a self-reported reduction of ≥ 50% in the average number of smoked cigarettes per day during week 21-24 compared to baseline, verified by a reduced concentration of carbon monoxide (CO) in exhaled air of at least 1 ppm. The protocol also included exploratory analyses of extent of smoking reduction (preceding 7 day period including abstinence days) compared to baseline according to predefined categories (100%, 75-99%, 50-74%, 25-49%, and < 25%).

Secondary end-points were evaluated at the week 12, 24, 36 and 48 clinical visits and included: CO-verified smoking reduction (≥ 50%) after 12 weeks (preceding 7 day period including abstinence days), point-prevalence estimate of smoking cessation (defined as self-reported total abstention from cigarettes during the preceding 7 day period verified by a concentration of CO in exhaled air of < 10 ppm at the clinical visit), estimates of continued smoking cessation (defined as self-reported total abstention from cigarettes during the preceding 4, 12, or 24-week period verified by a concentration of CO in exhaled air of < 10 ppm at all measurements during the specified period), and clinical tests and biomarkers including body weight, body mass index (BMI, defined as weight/height^2^), blood pressure, CO in exhaled air, pulmonary function tests including forced expiratory volume during one second (FEV_1.0_), forced vital capacity (FVC), the ratio between FEV_1.0 _and FVC (FEV%), and blood biomarkers (S-WBC, S-CRP, total S-cholesterol, S-HDL, S-LDL, S-fibrinogen, and S-cotinine).

### Adverse events

An adverse event (AE) was defined as any symptom, physical sign or disease that either emerged during the study or, if present at the baseline visit, worsened during the study, regardless of the suspected cause of the event. Each AE was described by the responsible trialist in terms of duration, frequency, intensity, association with the study medication, assessment of possible causes, actions taken, and outcome. AEs for which an association with the allocated study product was considered "possible", "probable", or "definite" are reported in this paper as treatment-related. A serious AE (SAE) was defined as any AE that was fatal or life-threatening, permanently disabling, resulting in unplanned or prolonged hospitalization, or if medical interventions were required to prevent any of the mentioned outcomes.

### Study monitoring and data handling

The study was coordinated and monitored by research personnel from an external contractor with a local office in Belgrade (i3 Research). They were also responsible for all data handling.

### Statistical analysis

Efficacy data and intent-to-treat comparisons are reported for all randomized participants. All statistical methods were based on the International Conference on Harmonization (ICH) E9 document "Statistical Principles for Clinical Trials" [20]. The statistical analyses were performed using SAS^® ^v9.2 for Windows by an external contractor (i3 Statprobe).

In order to reliably detect (p < 0.05, statistical power > 80%) a more than two-fold increase in the odds of achieving smoking reduction at 24 weeks among the snus versus placebo groups, and assuming a smoking reduction rate of 15% in the placebo group versus 28% in the snus group (corresponding to an odds ratio of 2.2), the target sample size was estimated at 156 per treatment group for a total size of 312 study participants.

Demographics, vital statistics and other clinical data were summarized using summary statistics for continuous variables or by way of group frequencies and percentages for categorical variables.

In the efficacy analyses participants with missing or incomplete information, typically because of non-compliance with follow-up visits, were counted as not having achieved the end-point in question. Intent-to-treat comparisons of the proportion of participants achieving smoking reduction or cessation were done using logistic regression techniques allowing for allocated treatment, center, age at baseline, gender, and the interaction between treatment and center. Odds ratios were computed along with the 95% confidence intervals (95% C.I.) and two-sided p-values. The exploratory analyses of level of smoking reduction in the two treatment groups were done using Pearson's chi-squared test. Changes over time of average number of cigarettes smoked, vital signs, and biomarker data were analyzed using mixed effects repeated measures models. The models included allocated treatment, center, age at baseline, gender, and the interaction between treatment and center as fixed effects, and used unstructured residual covariance matrices for repeated records within subjects.

## Results

### Participant disposition

A total of 319 participants entered the study during January, 2008 through April, 2009. The 48-week study completion rates were 56% (88/158) for the snus group, and 63% (101/161) for the placebo group (Table 1). Among the total of 130 participants who discontinued prematurely, the most common reasons in both treatment groups were failure to achieve the protocol definition of smoking reduction at the week 24 visit (57/130, 43.8%), withdrawal of informed consent (41/130, 31.5%), and loss to follow-up (21/130, 16.2%).

**Table 1:** Participant disposition

	319 Randomized	
Baseline:	158 (100%) Assigned to snus	161 (100%) Assigned to placebo

	158 (100%) Received assigned product, included in efficacy and safety analyses	161 (100%) Received assigned product, included in efficacy and safety analyses

Week 1-24:	26 (16%) Discontinued study	23 (14%) Discontinued study

	132 (84%) Completed protocol follow-up	138 (86%) Completed protocol follow-up

Week 24 visit:	31 (20%) Failed to achieve protocol definition of smoking reduction	29 (18%) Failed to achieve protocol definition of smoking reduction

	101 (64%) Continued in the study	109 (68%) Continued in the study

Week 24-48:	13 (8%) Discontinued study	8 (5%) Discontinued study

	88 (56%) Completed protocol follow-up	101 (63%) Completed protocol follow-up

Baseline and demographic characteristics were similar in the snus and placebo groups (Table 2). Overall, 61% were female. On average, participants were aged 44 years, had smoked 27 cigarettes per day during the past year, and had made 0.6 previous quit attempts. Most of them (81%) participated in the trial because they wanted to quit rather than just reduce their smoking. Few participants had previous exposure to nicotine replacement therapy (0.9%) or other pharmaceutical cessation aids (1.3%).

**Table 2:** Participant characteristics at baseline

Characteristic	Snus group (n = 158)	Placebo group (n = 161)
Age, mean (SD), years	43.3 (9.9)	44.0 (10.3)

Female (%)	99 (62.7)	97 (60.2)

Weight, mean (SD), kg	76.4 (16.5)	75.8 (16.4)

Height, mean (SD), cm	170.6 (8.8)	171.9 (10.0)

BMI (kg/m^2^), mean (SD)	26.1 (4.7)	25.5 (4.1)

Age at smoking initiation, mean (SD), years	19.2 (5.3)	18.8 (4.0)

Average no. of smoked cigarettes per day during last year, mean (SD)	27.6 (10.5)	25.7 (9.0)

No. of previous quit attempts, mean (SD)	0.7 (1.4)	0.6 (1.3)

Previous NRT exposure (%)	1 (0.6)	2 (1.2)

Previous exposure to other pharmaceutical smoking cessation products (%)	1 (0.6)	3 (1.9)

Intention to participate was to quit smoking (%)	132 (83.5)	127 (78.9)

FTND score, mean	6.2	6.1

SD: standard deviation, NRT: nicotine replacement therapy, FTND: Fagerström test for nicotine dependence

### Study product usage

**Figure 1:**
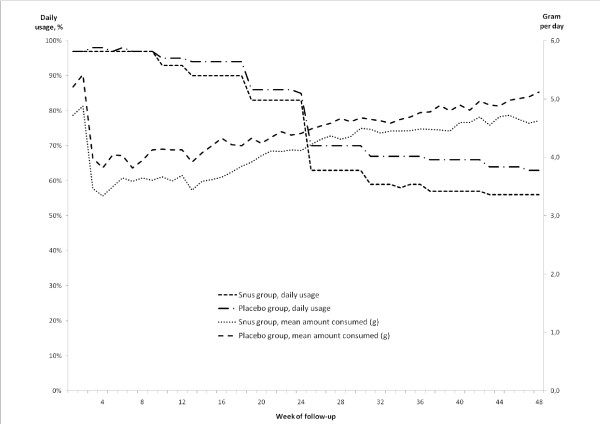
**Study product usage**. Proportion of participants reporting daily use (at least one sachet per day) of study product, and their mean daily consumption, by treatment allocation and week of follow up.

After the first week 97% of participants in both the snus and placebo groups reported some daily use of their allocated study product defined as having used at least one sachet per day during the preceding week. This proportion declined over time and was 52% after 48 weeks in the snus group compared to 60.2% in the placebo group (Figure 1). Among the daily users of snus the mean amount used per day was moderate: the weekly average ranged from 3.5 to 4.7 g per day, and was relatively stable over time. Those allocated to the placebo group had a marginally higher consumption. After the first few weeks c. 70-80% of those who reported daily product use preferred the small, 0.5 g sachets and the mean number of sachets used per day in both treatment groups was c.7-8. This number was similar irrespective of preferred sachet size.

### Cigarette consumption

**Figure 2:**
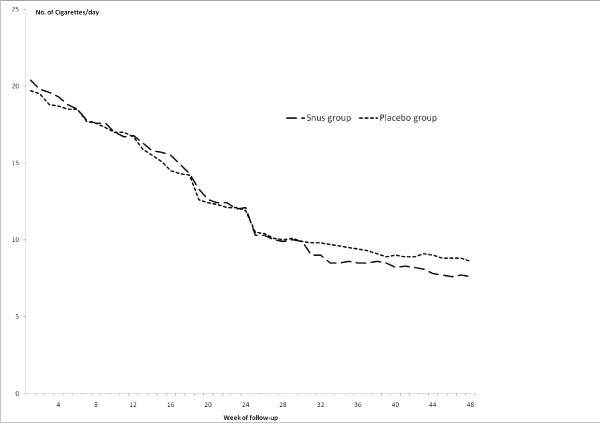
**Cigarette consumption**. Self-reported mean number of cigarettes smoked per day during the preceding week by treatment allocation and week of follow up.

The self-reported mean number of cigarettes smoked per day (including abstinence days) decreased over time in both the snus group and the placebo group (Figure 2, p < 0.001). Among those allocated to snus the decrease was slightly, but not statistically significantly more pronounced compared to the placebo group during week 30-48. At the week 48 visit the mean number in the snus group was 7.6 compared to 8.6 in the placebo group, that is, less than one third compared to baseline in both groups.

### Cotinine and CO in exhaled air

S-cotinine decreased substantially and similarly over time in both treatment groups (p < 0.001): at baseline the mean concentration in the snus and placebo group was 98.9 ng/mL and 101.2 ng/mL, respectively. The corresponding mean concentrations in the two groups during follow up were 70.9 and 70.6 (12 weeks), 68.7 and 71.7 (24 weeks), 62.9 and 69.3 (36 weeks), and 66.1 and 69.1 (48 weeks). Also, CO in exhaled air decreased statistically significantly over time (p < 0.001) in both treatment groups: at baseline the mean concentration was 23.5 ppm in both the snus and placebo group. The corresponding mean concentrations in the two groups during follow up were 20.0 and 20.2 (12 weeks), 16.7 and 15.8 (24 weeks), 13.0 and 13.2 (36 weeks), and 11.5 and 12.1 (48 weeks). The observed decreases of cotinine and CO in both treatment groups were thus less pronounced than the reported decreases in number of smoked cigarettes.

### Efficacy estimates

#### Smoking reduction

At the week 24 visit, a total of 101 participants (63.9%) in the snus group achieved a ≥ 50% smoking reduction according to the protocol definition compared to 109 (67.7%) in the placebo group. This difference was not statistically significant: the estimated odds ratio (snus versus placebo group) was 0.81 (95% C.I.: 0.48-1.36, p = 0.42). At the week 12 visit the corresponding proportions were 19.6% in the snus group (31/158) versus 12.4% in the placebo group (20/161) for an estimated odds ratio of 1.7 (95% C.I.: 0.94-3.23, p = 0.08).

The exploratory analyses of smoking reduction according to the predefined categories revealed that the proportion of participants reporting more extreme reductions in their average number of smoked cigarettes per day (≥ 75%) at the week 24 visit was statistically significantly higher (p < 0.01) in the snus group (15/158, 9.5%) than in the placebo group (4/161, 2.5%). At week 36 and 48 the corresponding proportions were 27/158 (17.1%) versus 17/161 (10.6%, p = 0.09), and 30/158 (19.0%) versus 21/161 (13.0%, p = 0.15).

#### Point-prevalence smoking abstinence

The number of participants with CO confirmed 7 day point prevalence abstinence was higher in the snus group compared to the placebo group at the clinical visits week 12, 24, 36, and 48. The estimated odds ratios (snus versus placebo group) ranged from 1.9 to 3.4, but only the estimate at 36 weeks was statistically significant (Table 3).

**Table 3:** CO-verified smoking cessation outcomes

Outcome	Snus, n = 158 (%)	Placebo, n = 161 (%)	Odds ratio (snus vs placebo)	**95% C.I**.	P
Point-prevalence cessation (1 week):					

-week 12	2 (1.3)	0	-	-	-

-week 24	9 (5.7)	3 (1.9)	3.4	0.9-12.8	0.08

-week 36	15 (9.5)	6 (3.7)	2.7	1.0-7.3	0.04

-week 48	25 (15.8)	15 (9.3)	1.9	0.9-3.7	0.08

Continued cessation at week 36:					

-4 weeks	13 (8.2)	6 (3.7)	2.3	0.9-6.4	0.10

-12 weeks	9 (5.7)	3 (1.9)	3.3	0.9-12.5	0.08

-24 weeks	2 (1.3)	0	-	-	-

Continued cessation at week 48:					

-4 weeks	22 (13.9)	12 (7.5)	2.1	1.0-4.4	0.06

-12 weeks	15 (9.5)	6 (3.7)	2.7	1.0-7.3	0.04

-24 weeks	9 (5.7)	3 (1.9)	3.3	0.9-12.5	0.08

#### Continuous smoking abstinence

The number of participants with CO confirmed smoking abstinence during the preceding 4, 12, and 24 week period was higher in the snus group compared to the placebo group at both the week 36 and week 48 visit. The estimated odds ratios ranged from 2.1 to 3.3, but only the estimate for 12-week continued abstinence at week 48 was statistically significant (Table 3)

### Baseline comparisons

#### Vital signs

Mean blood pressure (systolic and diastolic), body weight, BMI, and the tests for pulmonary function (FEV_1.0_, FVC, FEV%) did not change appreciably over time and there were no statistically significant differences between the two treatment groups (data not shown).

#### Biomarkers

The levels of S-WBC, S-CRP, total S-Cholesterol, S-HDL, S-LDL, and S-fibrinogen did not change appreciably over time and no statistically significant differences between the treatment groups were observed (data not shown).

#### Nicotine dependence

The average FTND score among those who continued to smoke was lower at the week 24 and 48 clinical visits compared to baseline but there was no difference between participants according to allocated treatment. In the snus group the average score at baseline, after 24 weeks, and 48 weeks was 6.2, 4.2, and 4.0, respectively. Among the placebo participants the corresponding scores were 6.1, 4.1, and 3.6.

The reported decrease in cigarette consumption among participants in both treatment groups contributed to the observed decreases in FTND as number of cigarettes smoked per day is one out of the six items in the instrument. It was beyond the scope of the current paper to perform an exploratory analysis of the contribution from the other items

### Safety and tolerability

Of the 319 participants all reported having used at least one sachet of their allocated study product and were consequently included in the safety analysis. Using snus was safe and generally well tolerated (Table 4). However, treatment-related AEs were reported by 30 participants allocated to snus (19.0%) compared to 18 in the placebo group (11.2%, p = 0.06), but they were mostly classified as mild and did not result in discontinuation of study treatment. Treatment-related AEs that occurred more frequently in the snus group typically concerned participants with symptoms related to nicotine exposure, such as nausea (17 participants in the snus group versus 12 in the control group), increased salivation (2 versus none), vomiting (2 versus none), and hiccups (1 versus none). Four participants in the snus group were also diagnosed with gingival or buccal irritation compared to one participant from the control group. However, none of these differences for specific AEs were statistically significantly different between the treatment groups.

**Table 4:** Summary of adverse events (AE)

	Snus group, n = 158 (%)	Placebo group, n = 161 (%)
Any AE	42 (26.6)	26 (16.1)

SAE	1 (0.6)	0

AE leading to discontinuation of study treatment	2 (1.3)	0

Treatment-related AE	30 (19.0)	18 (11.2)

SAE: serious adverse event, Treatment-related: relation to allocated study treatment considered by the trialist to be possible, probable, or definite,

One participant in the snus group developed an SAE (severe muscular weakness). It was classified as unrelated to use of study product but led to discontinuation of treatment. Another participant in the snus group discontinued using snus because of an AE (anxiety) which was also classified as unrelated to use of study product. No SAE was reported among the participants allocated to placebo.

## Discussion

The current results suggested that participants allocated to snus were more likely to quit smoking completely than participants allocated to placebo snus. Although the odds ratios at all time-points for the protocol-defined estimates of smoking cessation indicated that those allocated to snus were 1.9 to 3.4 times more likely to quit, the findings were of borderline significance with p-values ranging from 0.04-0.10.

The primary endpoint in the trial was a ≥ 50% smoking reduction at the week 24 visit. In contrast to complete cessation, the results indicated no difference between the snus and placebo groups in terms of this measure. The fact that the daily number of smoked cigarettes at baseline in both treatment groups was relatively high may help to explain this finding; the average participant only needed to decrease daily consumption to 13-14 cigarettes in order to fulfill the major protocol criteria for this end-point. However, the proportion of participants reporting more extreme decreases of the average number of cigarettes smoked per day (≥ 75% compared to baseline) was statistically significantly higher in the snus group compared to the placebo group at the week 24 visit (9.5% versus 2.5%, p < 0.01). For the average participant such reduction corresponded to daily smoking of less than c. 7 cigarettes per day. It has been suggested that a smoker needs to decrease consumption to low absolute levels to achieve beneficial effects on smoking-related morbidity as less extreme absolute reductions may be offset by compensatory smoking [21,22].

The main strength of this trial was the double-blind, placebo-controlled design although the protocol did not include procedures to assess the success of the blinding. The main weakness was that the study centers had not previously been involved in smoking cessation programs or worked with either pharmaceutical or behavioral cessation interventions. This may have contributed to the observed relatively low overall quit rate. Another contributing factor may have been that the social environment in Serbia, with a high smoking prevalence, few smoking restrictions, and a generally low public awareness of the dangers of smoking, is not supportive of quit attempts among smokers who want to stop smoking. An illustration to this was perhaps the low mean number of previous quit attempts among the participants (Table 2).

Nicotine is not harmless but it is the inhalation of combustion products accompanying the nicotine in tobacco smoke that explains most of the excess morbidity and mortality experienced by smokers. These circumstances formed part of the rationale for the study design which included usage of study product *ad libitum *over the entire 48 week study period with no prescribed tapering of product use after a specified time point. The aims of the trial thus did not include treating the participants' nicotine dependence. Clinical experience from Scandinavia indicates that smokers who use snus as a smoking cessation aid typically do not switch abruptly from cigarettes to snus. The transition period of dual daily use can last from weeks to months, so in this study there was a grace period of 24 weeks before the participants were actively instructed to completely refrain from smoking. On the other hand, a long "grace period" before a target quit date could theoretically lead to dissipation of the motivation to quit among some smokers. Smokers who have successfully quit by switching to snus typically do not abruptly stop using snus after a few weeks or months. In fact, a substantial proportion of smokers who have switched to snus become long term users [23]. The same appears to apply also to pharmaceutical nicotine; several studies have indicated that a proportion of ex-smokers who quit using NRT continue to use such products long-term [24-26].

Beneficial effects from smoking reduction or cessation on vital signs (e.g. blood pressure and pulmonary function) and biomarker levels (e.g. CRP, fibrinogen, and blood lipids) are mainly observed among those who quit completely and typically take several weeks to months to emerge. The overall low complete cessation rates in this study may have contributed to the fact that we could not detect any statistically significant overall differences between the treatment groups in terms of such measures, despite the difference in number of quitters in favor of the snus group. Any differences that may have occurred as a result of this difference were probably obscured by the results for the large number of non-quitters. The generally small number of quitters also precluded meaningful exploratory analyses according to quitting behavior.

Unassisted cessation remains the most common method by which smokers quit, but in Scandinavia snus is the most frequently reported method among those who use some form of cessation aid [27,28]. Also, snus appears to be associated with a higher long-term success rate compared to pharmaceutical nicotine [28]. Possible explanations include the more rapid nicotine delivery from snus [17], and the fact that snus is typically used more long term [23].

## Conclusions

Snus use is traditional in Sweden, particularly among males. It has been hypothesized that cultural factors may make snus unacceptable or ineffective as a smoking cessation aid outside of Scandinavia [29]. This trial demonstrated that Swedish snus was acceptable and could promote smoking cessation also among smokers in Serbia, that is, in a cultural setting without traditional use of any form of oral tobacco.

## Competing interests

The trial was officially sponsored by Swedish Match AB, Stockholm, Sweden. Sponsor provided funding, study products (snus and placebo snus), and study equipment. External contractors paid by the sponsor provided monitoring, data handling, and all statistical analyses (i3 Research, i3 Statprobe).

LER is an employee of Swedish Match AB.

GJ, VST, RA, and RN received honoraria from Swedish Match AB for their work with this trial, but declare no other conflict of interest.

## Authors' contributions

GJ participated in the design of the study, was responsible for coordination of trial activities, and helped draft the manuscript. VST was responsible for trial procedures at one of the participating study centers. RA was responsible for the pilot study, participated in the design of the trial and was responsible for coordination of trial activities and trial procedures. RN conceived of the study, participated in the trial design, coordinated trial activities, and helped draft the manuscript. LER participated in the trial design, was responsible for study product logistics, and drafted the manuscript. All authors critically revised the manuscript for important intellectual content, read and approved the final version.
